# Improving Outcomes and Quality of Life for Patients With Hand and Foot Eczema: Randomized Study of a Patient-Centered Monitoring App

**DOI:** 10.2196/52159

**Published:** 2025-01-21

**Authors:** Aimee Bruch, Wanja Weigandt, Yannic Schardt, Raphael Herr, Johannes Benecke, Astrid Schmieder

**Affiliations:** 1 Department of Dermatology, Venerology and Allergology University Medical Center Mannheim, Heidelberg University University Medical Center Mannheim, Heidelberg University Mannheim Germany; 2 Department of Medical Informatics, Biometry and Epidemiology Friedrich-Alexander-Universität Erlangen-Nürnberg Friedrich-Alexander-Universität Erlangen-Nürnberg Erlangen Germany; 3 Department of Dermatology, Venereology and Allergology University Hospital Würzburg Würzburg Germany

**Keywords:** hand and foot eczema, eHealth, mHealth, teledermatology, telemedicine, disease management, smartphone application, mental health, eczema, clinical outcome, quality of life, dermatology, pain, motor skills, educational, support, mobile phone

## Abstract

**Background:**

Hand and foot eczema is a frequent chronic dermatological condition. The persistent itching, pain, and blistering can impair hand and foot function, leading to difficulties in performing tasks requiring fine motor skills. In addition, the impact on the quality of life for affected patients is significant, as the symptoms can be extremely uncomfortable and disruptive to daily activities. By incorporating digital health apps and educational programs into the management of hand and foot eczema, patients may receive ongoing support, optimize their clinical outcomes, and ultimately enhance their overall quality of life.

**Objective:**

The purpose of this study was to evaluate the effect of a smartphone app combined with educational training on the clinical outcomes and mental health of patients with chronic hand and foot eczema during a 60-week study period.

**Methods:**

Patients in the intervention group participated in an educational program focused on chronic hand and foot eczema at baseline and had in-person visits at weeks 0, 12, 24, 36, and 60, as well as access to our study smartphone app. The app allowed patients to upload pictures of their hands and feet and answer questions about pain severity, itching, mood, and quality of life. A chat function was also available for patients to contact their dermatologist. The control group received only the in-person study visits described above.

**Results:**

A total of 87 patients were included in the study and randomized to the intervention (n=43) or control (n=44) groups. In total, 23 patients from the intervention group and 34 patients from the control group completed the study. Throughout the 60-week study period, a significant reduction in Hand Eczema Severity Index (HECSI) was consistently observed in all patients (week 60: linear regression coefficient [Coef]=–1.108; *P*≤.001). A trend toward a greater improvement of the HECSI in the intervention group compared to the control group was noticed (week 60: Coef=0.597; *P*=.05). Subgroup analysis revealed that patients who used the app with a usage frequency of less than 20% demonstrated a significant reduction in the HECSI from week 0 to week 60 (week 60: Coef=–1.275; *P*=.04) and a significant reduction in the Dermatology Life Quality Index (week 60: Coef=–1.246; *P*=.04) compared to the control group. We were able to demonstrate a significant correlation between the HECSI calculated based on pictures uploaded by patients through the app and the HECSI assessed during personal visits (ρ=0.885; *P*<.001), despite the potentially lower image quality of the pictures uploaded through the app.

**Conclusions:**

This study provides further evidence that digital health apps can provide valuable support in improving patient clinical outcomes and management, especially as the app-based assessment of hand and feed images appears to be reliable.

**Trial Registration:**

Deutsches Register Klinischer Studien DRKS00020963; https://drks.de/search/de/trial/DRKS00020963

## Introduction

Hand eczema is one of the most common dermatologic conditions responsible for a very large proportion of dermatological consultations. According to a systematic review and meta-analysis, the 1-year prevalence is estimated to be 9.1% in the general population [1].

The probability of developing hand eczema at least once in a lifetime is 17% [2]. This likelihood varies greatly depending on occupational groups. Occupations that involve a lot of manual labor or chronic exposure to mild toxins or irritants, such as hairdressers and bricklayers, have a higher chance of developing hand eczema at least once in their lifetime [2]. The incidence of the disease is currently on the rise due to improved hygiene standards, increased life expectancy, and more frequent atopic predisposition [2].

Hand eczema is considered chronic if it lasts longer than 3 months or is recurrent more than twice a year [3]. Clinical manifestations are usually highly variable. In the acute stage, macules, papules, edema, vesicles, or even bullae may appear, whereas in the chronic stage, scales and crusts, hyperkeratosis, rhagades, and lichenification prevail [4]. These skin lesions can cause itching, burning, and pain, thereby leading to sleep disturbances and mood changes [5]. Chronic hand eczema is of high health-economic and sociomedical importance, as this diagnosis is responsible for many cases of prolonged sick leave and avoidance of social and public life [4,6]. In particular, the hands are an important organ of communication that is difficult to conceal from the public. Stigmatization thus causes significant psychosocial distress and a reduction in quality of life [7,8].

As hand and foot eczema is an intermittent disease, it is often our clinical experience that patients miss the optimal time to seek medical attention. Appointments are frequently not available on short notice, leading to patients presenting with very vigorous symptoms. This highlights the need for an easily accessible alternative for people with chronic hand and foot eczema such as a digital health app.

Germany recently launched the directory for Digital Health Applications (DiGA), which lists Conformité Européenne–marked medical devices. These apps are approved by the Federal Institute for Drugs and Medical Devices and are designed to improve patient outcomes through digital means, offering support in managing medical conditions, tracking symptoms, and providing tailored care plans. Such DiGA can be prescribed by the doctor and are paid by German health insurance companies. Although chronic hand and foot eczema is a dermatological disease easy to monitor through pictures taken by the patients, there are currently no specific DiGA available that offer support for these patients [9]. Furthermore, to the best of our knowledge, scientific data on the beneficial effects of DiGA for patients with hand and foot eczema is limited. This lack of evidence highlights a critical need for further research, especially given the prevalence of these conditions.

We developed a monitoring app for patients with chronic hand and foot eczema, enabling them to regularly photograph their condition and answer disease-specific questions. The app also facilitated teledermatology appointments tailored to individual patient needs.

To evaluate the effectiveness of this tool for chronic hand and foot eczema, we conducted an interventional study comparing a control group (without the app or educational program) with an intervention group over a 60-week period. Interim results from a 24-week analysis already showed an improvement in quality of life [10].

The main objective of the 60-week study was to assess the impact of regular physician-patient interactions and a prestudy educational program on disease progression, quality of life, and overall patient outcomes over the long-term period of 60 weeks.

## Methods

### Study Design

This intervention study was undertaken at the Department of Dermatology, Venerology and Allergology at the University Medical Center Mannheim, Germany, between August 2018 and August 2021. This is the analysis of the data from the study weeks 0 (V0), 12 (V2), 24 (V3), 36 (V4), and 60 (V5). The inclusion criteria that had to be fulfilled by the patients in order to be eligible to participate in the study were (1) a physician-confirmed diagnosis of chronic hand and foot eczema, (2) possession of a smartphone and being able to use it, (3) an age between 18 and 75 years, as well as (4) the ability to provide informed consent. Exclusion criteria were the inability to provide informed consent and being younger than 18 years old or older than 75 years old. During the first study visit (V0), patients were randomly assigned to the control or intervention group in a 1:1 ratio by shuffling a deck of cards.

A total of 90 patients were included in the study. Of the total, 43 patients were in the intervention group, 44 in the control group, and 3 patients dropped out from the study before being assigned to the respective group.

The control group started the first visit at week 0. At the first visit, a detailed medical history including sociodemographic data and eczema-related data were collected. In addition, the Dermatology Life Quality Index (DLQI; 0-30) was assessed and the Hand Eczema Severity Index (HECSI; 0-36) was calculated. Using a numerical rating scale (0-10), each patient was asked how much the eczema negatively affects activities and mood, and how much the eczema currently itches or hurts. Face-to-face follow-up was performed at weeks 12 (V2), 24 (V3), 36 (V4), and 60 (V5).

In addition to this first face-to-face visit, the intervention group participated in an educational program. This 2-hour educational session provided patients with information on the etiology, pathogenesis, exacerbating factors, and treatment of chronic hand and foot eczema. It was conducted by experienced specialists in dermatology (AS and JB). The patients had the opportunity to exchange information with specialists. In addition, each patient in the intervention group received a personal anonymized access code to our app, Dermascope Mobile (DermaIntelligence GmbH), as well as instructions on how to use it. Screenshots of the app can be found in the article by Domogalla et al [11].

Participants were asked to take pictures of their eczema once a week through the app and answer questions about their current state of health, including their psyche, and complete questionnaires on quality of life (DLQI) and current symptoms. The app also had a chat function with which patients could contact their treating dermatologist. The app could not be used more than once a week.

Each image uploaded through the app was classified by us into good or bad quality depending on the sharpness of the image, the light conditions, and whether you could see the whole hand or foot or not. An image could only be rated as good if every criterion was positively evaluated. Based on these images, we calculated the electronic HECSI (eHECSI), which we then compared to the HECSI assessed in person.

### Ethical Considerations

The Medical Ethics Committee of the Medical Faculty Mannheim, Heidelberg University approved the study (ethics approval 2017-655N-MA), and the implementation complied with the Helsinki Declaration. Informed consent was obtained from all participants before the study, and in the case of secondary data analysis, the original consent allowed for such use without requiring additional permissions. To protect privacy and confidentiality, all data were deidentified, with access restricted to authorized personnel. Participants received no compensation except from a good medical care, and no identifying information or images of individuals were included. The CONSORT-EHEALTH (Consolidated Standards of Reporting Trials of Electronic and Mobile Health Applications and Online Telehealth) checklist can be found in Multimedia Appendix 1.

### Statistical Analysis

Linear panel data regression analyses estimated outcome trajectories. Linear regression coefficients (Coef) used throughout the text describe the mathematical relationship between each independent and dependent variable, while *P* values indicate whether these relationships are statistically significant. Random effects regressions determined the main and interaction effects of group membership (intervention vs control) and visit time (V1, V2, V3, V4, and V5) on DLQI, pain, activities of daily living, and HECSI scores. In total, 2 adjustment models were calculated. The first model was unadjusted, whereas the second model was adjusted for sex, age, and disease duration. Additional analyses included the effect of app usage frequency over 60 weeks (group membership: control vs <20% app usage frequency vs ≥20% app usage frequency). Therefore, the intervention group was divided into 2 groups—one consisting of patients with app use frequency <20% and the other consisting of patients with app use frequency ≥20% during the 24-week observation period. The chosen cutoff of 20% corresponds to an app use frequency of once every 5 weeks. Variables were tested for normal distribution and, where appropriate, transformed to approximate normal distribution (power transformation of the square root of the DLQI and log10 of the HECSI). All statistical analyses were performed with Stata Special Edition (version 14.0; StataCorp).

## Results

### Patient Demographics

**Figure 1:**
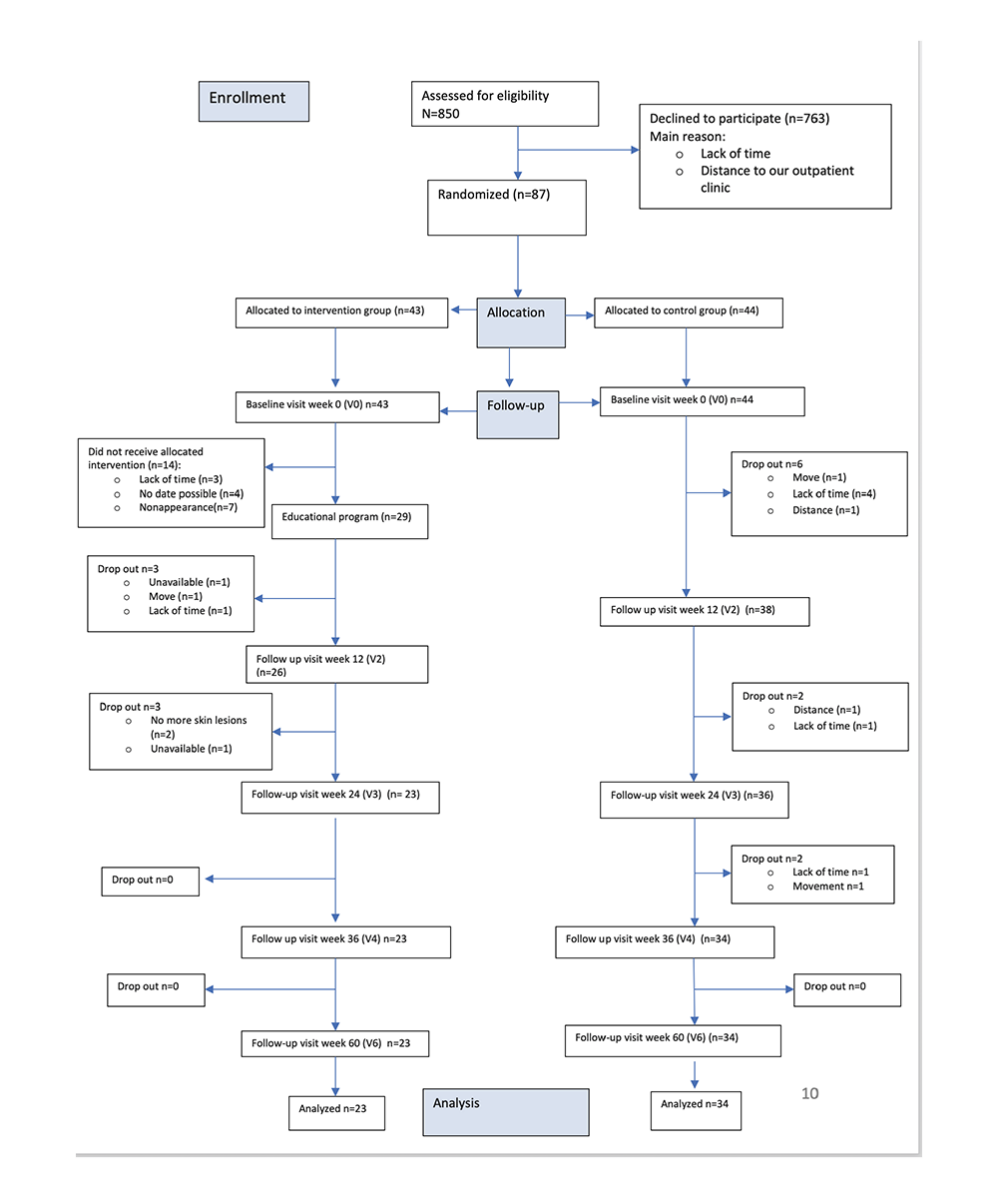
Flow diagram of the study cohort.

Of the 90 patients included in the study, 43 patients were in the intervention group and 44 in the control group (Table 1, Figure 1). In addition, 3 patients dropped out of the study before being assigned to the respective group. Furthermore, 57 patients completed the study and could be included in the final analysis, 23 patients were from the intervention group, and 34 were from the control group. The most common reasons for withdrawal were lack of time, improvement of local findings, or long distance to the clinic.

**Table 1:** Patient characteristics at week 0 (V0) adapted from Weigandt et al [10].

Characteristics		Overall (n=87)	Control (n=44)	Intervention (n=43)
**Sex, n (%)**				
	Female	51 (59)	25 (57)	26 (60)
	Male	36 (41)	19 (43)	17 (40)
**Age (years)**				
	Mean (SD)	47.07 (15.42)	48.05 (14.09)	46.07 (16.78)
	Median	50	51	49
**BMI (kg/m^2^)**				
	Mean (SD)	27.62 (7.53)	26.45 (5.27)	28.82 (9.2)
	Median	2578	25.16	26.81
Smoker, n (%)		29 (33)	16 (36)	13 (30)
**Duration of eczema (years)**				
	Mean (SD)	6.9 (8.23)	6.0 (8.47)	7.81 (7.98)
	Median	4	3	6
**HECSI^a^ (range 0-360)**				
	Mean (SD)	22.53 (21.29)	20.93 (20.72)	24.16 (21.99)
	Median	18	15	19
**DLQI^b^ (range 0-30)**				
	Mean (SD)	7.97 (6.38)	7.73 (7.16)	8.21 (5.55)
	Median	6	6	8
**Pain (range 0-10)**				
	Mean (SD)	1.94 (2.67)	2.14 (2.77)	1.74 (2.59)
	Median	0	1	0
**Activity (range 0-10)**				
	Mean (SD)	4.02 (3.23)	3.95 (3.37)	4.09 (3.12)
	Median	4	4	4

^a^HECSI: Hand Eczema Severity Index.

^b^DLQI: Dermatology Life Quality Index.

### Comparison of Clinical Outcomes Between the Intervention and Control Groups

**Figure 2:**
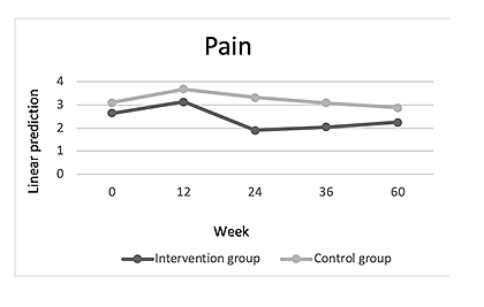
Comparison of pain development between intervention and control groups over the 60-week study period (observant=290; n=57).

At the end of the 60-week study period, there was a reduction of pain in both groups compared to their baseline visits (week 60: Coef=–0.400; *P*=.53; Table 2, model 0). There were no significant differences between the control group and the intervention group (week×group: week 60: Coef=–0.175; *P*=.83; Table 2, model 0; Figure 2). The greatest reduction in pain in both groups was noted between week 12 and 24 (week 12: Coef=–0.478; *P*=.44; week 24: Coef=–0.739; *P*=.24; week 36: Coef=–0.608; *P*=.33; week 60: Coef=–0.400; *P*=.53; Table 2, model 0; Figure 2).

**Table 2:** Random effect regression models over 60 weeks; model 0 unadjusted; model 1 adjusted for age, sex, and disease duration (n= 57; observations=290).

Regression models for pain, activity, HECSI^a^, and DLQI^b^				Model 0					Model 1		
				Linear regression coefficient (SE)		*P* value		Linear regression coefficient (SE)			*P* value
**Pain**											
	**Week**										
		0	Reference value		Reference value		Reference value			Reference value	
		12	–0.478 (0.622)		.44		–0.478 (0.622)			.44	
		24	–0.739 (0.622)		.24		–0.739 (0.622)			.24	
		36	–0.608 (0.622)		.33		–0.608 (0.622)			.33	
		60	–0.400 (0.631)		.53		–0.386 (0.630)			.54	
	**Week×group**										
		0**×**control	Reference value		Reference value		Reference value			Reference value	
		12**×**control	–0.105 (0.796)		.90		–0.105 (0.796)			.90	
		24**×**control	–0.961 (0.796)		.23		–0.961 (0.796)			.23	
		36**×**control	–0.590 (0.802)		.46		–0.578 (0.802)			.47	
		60**×**control	–0.175 (0.808)		.83		–0.150 (0.808)			.85	
**Activity**											
	**Week**										
		0	Reference value		Reference value		Reference value			Reference value	
		12	–1.391 (0.638)		.03		–1.391 (0.641)			.03	
		24	–2.347 (0.638)		<.001		–2.347 (0.641)			<.001	
		36	–2.652 (0.638)		<.001		–2.652 (0.641)			<.001	
		60	–2.712 (0.647)		<.001		–269 (0.650)			<.001	
	**Week×group**										
		0**×**control	Reference value		Reference value		Reference value			Reference value	
		12**×**control	1.085 (0.817)		.18		1.085 (0.821)			.19	
		24**×**control	0.986 (0.817)		.23		0.986 (0.821)			.23	
		36**×**control	0.975 (0.823)		.24		0.957 (0.827)			.25	
		60**×**control	1.183 (0.829)		.15		1.149 (0.834)			.17	
**Mood**											
	**Week**										
		0	Reference value		Reference value		Reference value			Reference value	
		12	–0.913 (0.642)		.16		–0.913 (0.643)			.16	
		24	–1.652 (0.642)		.01		–1.652 (0.643)			.01	
		36	–1.782 (0.642)		.005		–1.782 (0.643)			.006	
		60	–1.924 (0.650)		.003		–1.911 (0.651)			.003	
	**Week×group**										
		0**×**control	Reference value		Reference value		Reference value			Reference value	
		12**×**control	0.051 (0.821)		.95		0.051 (0.823)			.95	
		24**×**control	0.541 (0.821)		.51		0.541 (0.823)			.51	
		36**×**control	–0.151 (0.827)		.86		–0.159 (0.829)			.85	
		60**×**control	–0.009 (0.834)		.99		–0.029 (0.835)			.97	
**HECSI**											
	**Week**										
		0	Reference value		Reference value		Reference value			Reference value	
		12	–0.512 (0.237)		.03		–0.512 (0.237)			.03	
		24	–0.715 (0.237)		.003		–0.715 (0.237)			.003	
		36	–0.873 (0.237)		<.001		–0.872 (0.237)			<.001	
		60	–1.108 (0.240)		<.001		–1.090 (0.240)			<.001	
	**Week×group**										
		0**×**control	Reference value		Reference value		Reference value			Reference value	
		12**×**control	0.327 (0.304)		.28		0.327 (0.304)			.28	
		24**×**control	0.428 (0.304)		.16		0.428 (0.304)			.16	
		36**×**control	0.509 (0.306)		.10		0.498 (0.306)			.10	
		60**×**control	0.597 (0.308)		.05		0.567 (0.308)			.07	
**DLQI**											
	**Week**										
		0	Reference value		Reference value		Reference value			Reference value	
		12	–0.560 (0.210)		.008		–0.560 (0.211)			.008	
		24	–0.855 (0.210)		<.001		–0.855 (0.211)			<.001	
		36	–1.105 (0.210)		<.001		–1.105 (0.211)			<.001	
		60	–1.152 (0.213)		<.001		–1.153 (0.214)			<.001	
	**Week×group**										
		0**×**control	Reference value		Reference value		Reference value			Reference value	
		12**×**control	0.450 (0.269)		.10		0.450 (0.271)			.10	
		24**×**control	0.420 (0.269)		.12		0.420 (0.271)			.12	
		36**×**control	0.710 (0.271)		.009		0.707 (0.272)			.01	
		60**×**control	0.553 (0.274)		.04		0.551 (0.275)			.05	

^a^HECSI: Hand Eczema Severity Index.

^b^DLQI: Dermatology Life Quality Index.

**Figure 3:**
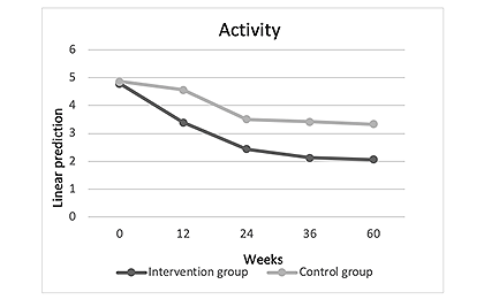
Activity development in the intervention group compared with the control group over the 60-week stud period (observant=290; n=57).

In terms of activity, we were able to demonstrate a significant reduction in impairment regardless of group membership (week 60: Coef=–2.712; *P*≤.001; Table 2, model 0; Figure 3). No significant differences or changes in the course and development were observed between the control and intervention group (week 60: Coef=1.183; *P*=.15; Table 2, model 0; Figure 3).

**Figure 4:**
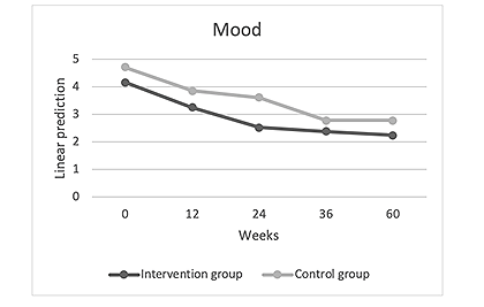
Mood development in the intervention group compared with the control group over the 60-week study period (observant=219; n=57).

In addition, the mood of patients with hand and foot eczema was ameliorated in both study groups. Significant reductions in scores were observed in nearly all subsequent visits compared to baseline without significant differences between both groups (week 12: Coef=–0.913; *P*=.16; week 24: Coef=–1.652; *P*=.01; week 36: Coef=–1.782; *P*=.005; week 60: Coef=–1.924; *P*=.003; Table 2, model 0; Figure 4).

**Figure 5:**
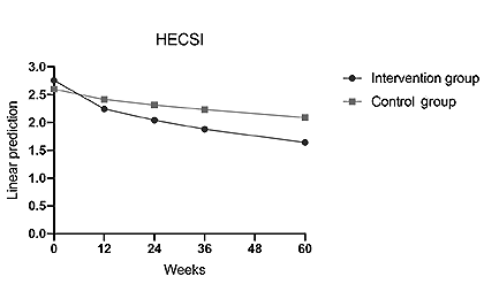
HECSI development in the intervention group compared to the control group over the 60-week study period (observant=219; n=57). HECSI: Hand Eczema Severity Index.

A significant reduction in HECSI scores was consistently observed across all patients over the course of 60 weeks, indicating substantial improvement compared to the initial assessment at week 0 (week 12: Coef=–0.512; *P*=.03; week 24: Coef=–0.715; *P*=.003; week 36: Coef=–0.873; *P*<.001; week 60: Coef=–1.108; *P*≤.001; Table 2, model 0; Figure 5). We observed a trend of greater improvement in the HECSI over time in the intervention group compared to the control group (week×control: week 36: Coef=0.509; *P*=.10; week 60: Coef=0.597; *P*=.05; Table 2, model 0; Figure 5).

**Figure 6:**
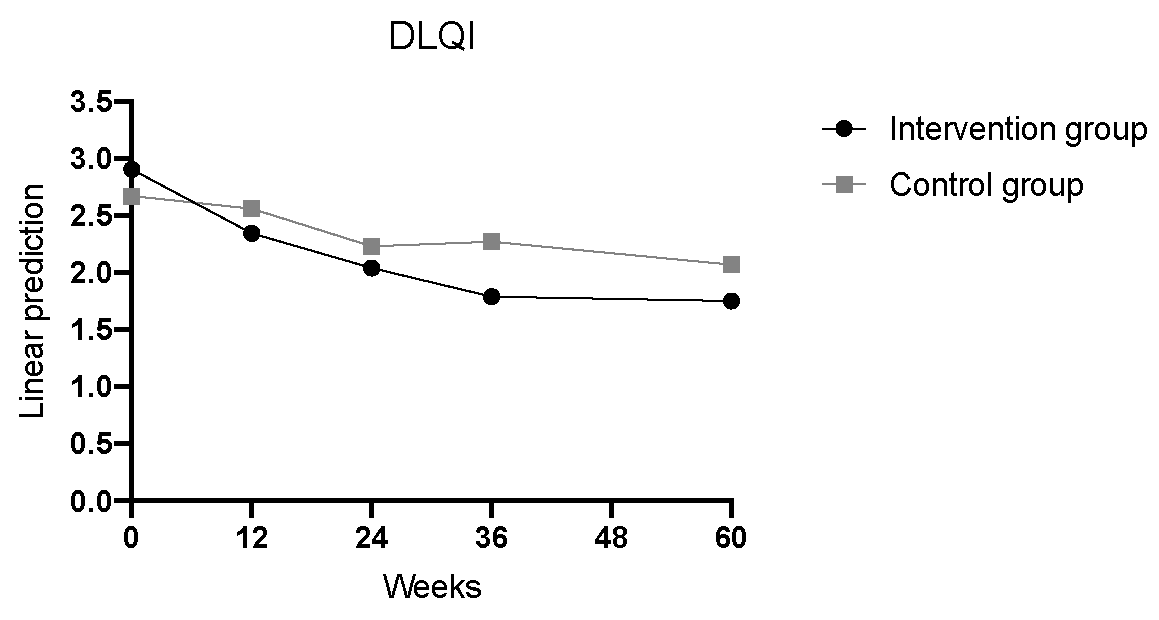
DLQI development in the intervention group compared to the control group over the 60-week study period (observant=219; n=57). DLQI: Dermatology Life Quality Index.

A significant reduction in the DLQI was detected at any time in all patients (week 12: Coef= –0.560; *P*=.008; week 24: Coef=–0.855; *P*≤.001; week 36: Coef=–1.105; *P*≤.001; week 60: Coef=–1.152; *P*≤.001; Table 2, model 0; Figure 6). Starting from week 36, a notable and statistically significant improvement was noted in the intervention group compared to the control group (week×control: week 24: Coef=0.420; *P*=.12; week 36: Coef= –0.710; *P*=.009; week 60: Coef=0.553; *P*=.04; Table 2, model 0; Figure 6).

The findings did not differ for sex, age, and disease duration (matching coefficients and *P* values in Table 2, model 1).

### App Usage Frequency Subgroup Analysis

For subgroup analysis, the intervention group was divided into 2 groups with patients using the app more or less frequently than once every 5 weeks, which equals an app use frequency of more or less than 20%. The frequency of app usage is measured solely by the actions of uploading images and completing the associated questionnaires. Messages sent to us through the chat function are not included in this count. The ability to ask questions and send messages was unrestricted.

Based on our data, the app usage frequency is influenced by gender (*P*=.006; Table 3) and age (*P*=.03; Table 3). Disease severity (HECSI) and itching had no impact on the frequency of app usage (Table 3). Women did use the app more often than men (women: 42/51, 82% vs men: 29/50, 58%; Table 3), and older women did use the app the most (mean age of women using the app more than 20%=54.787, SD 14.827 years and mean age of men using the app more than 20%=46.833, SD 15.099 years Table 4).

**Table 3:** Logistic regression of the app usage frequency subgroups ≥20% over 60 weeks (observant=101).

App frequency ≥20%	Odds ratio (95% CI)	SE	*P* value
Gender	4.309 (1.517-12.236)	2.294	.006
Age (V0^a^)	1.041 (1.004-1.081)	0.019	.03
HECSI^b^	1.005 (0.609-1.657)	0.256	.99
Itching	0.812 (0.636-1.036)	0.101	.10

^a^The age refers to the age at the time of the first visit (V0).

^b^HECSI: Hand Eczema Severity Index.

**Table 4:** Two sample t test with equal variances that show which average age (V0) has used the app the most among those people with a frequency more than 20% app usage.

Age	Observants	Mean^a^ (SD)	SE	95% CI
Men	30	46.833 (15.099)	2.756	41.194-52.471
Women	71	54.787 (14.827)	1.759	51.279-58.298
Combined	101	52.425 (15.276)	1.520	49.41-55.44

^a^Mean: mean age.

**Figure 7:**
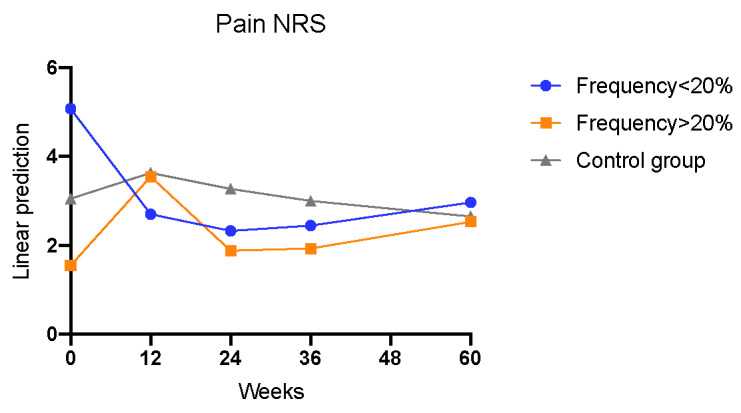
Development of the pain comparing the control to the intervention group (observant=290; n=57; interaction week×<20%: week 12: *P*=.009; week 24: *P*=.009; week 36: *P*=.009). NRS: numerical rating scale.

A significant reduction in terms of pain was assessed in patients using the app less than 20%, at weeks 12 and 24 compared to baseline, but this effect did not persist until week 60 when compared to the controls (interaction week×<20%; week 12: Coef=–2.958; *P*=.009; week 24: Coef=–2.972; *P*=.009; week 36: Coef=–2.581; *P*=.06; week 60: Coef=–1.711; *P*=.34; Table 5, model 1; Figure 7). Patients with an app usage frequency of more than 20% showed no significant reduction in pain over the study period (Table 5, model 1; Figure 7). Results were dependent on gender, age, and disease duration (Table 5, model 1).

**Table 5:** Random effect regression models of the app usage frequency subgroups <20% and ≥20% over 60 weeks. Model 0 is unadjusted, and model 1 is adjusted for age, sex, and disease duration (n=57; observants=290).

Regression models of the app usage frequency subgroups for pain, activity, mood, itching, HECSI^a^, and DLQI^b^			Model 0			Model 1	
			Linear regression coefficient (SE)	*P* value	Linear regression coefficient (SE)		*P* value
**Pain**							
	**Week×group**						
		0×control	Reference value	Reference value	Reference value		Reference value
		12×<20%	–3.069 (1.190)	.01	–2.958 (1.138)		.009
		12×≥20%	–0.278 (0.936)	.98	1.417 (0.895)		.11
		24×<20%	–1.764 (1.190)	.14	–2.972 (1.138)		.009
		24×≥20%	–0.572 (0.936)	.54	–0.111 (0.894)		.90
		36×<20%	–2.576 (1.452)	.08	–2.581 (1.389)		.06
		36×≥20%	–0.184 (0.933)	.84	0.468 (0.892)		.60
		60×<20%	–3.048 (1.862)	.10	–1.711 (1.782)		.34
		60×≥20%	–0.132 (1.025)	.90	1.384 (0.980)		.16
**Activity**							
	**Week×group**						
		0×control	Reference value	Reference value	Reference value		Reference value
		12×<20%	–1.416 (1.081)	.19	–3.069 (1.196)		.01
		12×≥20%	–0.133 (0.850)	.88	–0.0277 (0.940)		.98
		24×<20%	–0.555 (1.081)	.61	–1.764 (1.196)		.14
		24×≥20%	–0.355 (0.850)	.68	–0.572 (0.940)		.54
		36×<20%	–1.078 (1.323)	.42	–2.541 (1.459)		.08
		36×≥20%	0.439 (0.848)	.61	–0.128 (0.936)		.89
		60×<20%	–1.395 (1.701)	.41	–2.955 (1.869)		.11
		60×≥20%	–0.931 (0.933)	.32	–0.092 (1.029)		.93
**Mood**							
	**Week×group**						
		0×control	Reference value	Reference value	Reference value		Reference value
		12×<20%	–0.990 (0.444)	.03	–1.888 (1.197)		.12
		12×≥20%	0.026 (0.349)	.94	0.927 (0.941)		.32
		24×<20%	–0.651 (0.444)	.14	–1.388 (1.197)		.25
		24×≥20%	–0.310 (0.349)	.38	–0.088 (0.941)		.93
		36×<20%	–1.289 (0.542)	.02	–1.214 (1.464)		.41
		36×≥20%	–0.316 (0.348)	.36	0.823 (0.938)		.38
		60×<20%	–0.280 (0.696)	.69	–1.894 (1.881)		.31
		60×≥20%	–0.117 (–383)	.76	0.796 (1.032)		.44
**Itching**							
	**Week×group**						
		0×control	Reference value	Reference value	Reference value		Reference value
		12×<20%	–1.888 (1.197)	.11	–1.416 (1.081)		–1.31
		12×≥20%	0.927 (0.941)	.32	–0.133 (0.850)		.88
		24×<20%	–1.388 (1.197)	.25	–0.555 (1.081)		.61
		24×≥20%	–0.088 (0.941)	.93	–0.355 (0.850)		.68
		36×<20%	–1.210 (1.462)	.41	–1.044 )1.324)		.43
		36×≥20%	0.794 (0.938)	.40	0.460 (0.843)		.59
		60×<20%	–1.926 (1.878)	.31	–1.320 (1.703)		.44
		60×≥20%	0.777 (1.032)	.45	0.941 (0.933)		.31
**HECSI**							
	**Week×group**						
		0×control	Reference value	Reference value	Reference value		Reference value
		12×<20%	–1.234 (0.3916)	.002	–0.990 (0.445)		.03
		12×≥20%	–0.032 (0.307)	.92	0.026 (0.350)		.94
		24×<20%	–0.732 (0.391)	.06	–0.651 (0.445)		.11
		24×≥20%	–0.253 (0.307)	.41	–0.310 (0.350)		.38
		36×<20%	–1.500 (0.479)	.002	–1.217 (0.542)		.03
		36×≥20%	–0.480 (0.307)	.12	–0.284 (0.348)		.41
		60×<20%	–1.275 (0.616)	.04	–0.196 (0.693)		.78
		60×≥20%	–0.133 (0.338)	.69	–0.077 (0.382)		.84
**DLQI**							
	**Week×group**						
		0×control	Reference value	Reference value	Reference value		Reference value
		12×<20%	–0.081 (0.319)	.79	–1.234 (0.392)		.002
		12×≥20%	0.093 (0.251)	.71	–0.032 (0.309)		.92
		24×<20%	0.278 (0.319)	.38	–0.732 (0.393)		.06
		24×≥20%	0.155 (0.251)	.54	–0.253 (0.309)		.41
		36×<20%	0.652 (0.391)	.10	–1.485 (0.481)		.002
		36×≥20%	0.261 (0.250)	.30	–0.465 (0.038)		.13
		60×<20%	0.995 (0.504)	.05	–1.246 (0.619)		.04
		60×≥20%	0.329 (0.276)	.23	–.127 (0.339)		.71

^a^HECSI: Hand Eczema Severity Index.

^b^DLQI: Dermatology Life Quality Index.

**Figure 8:**
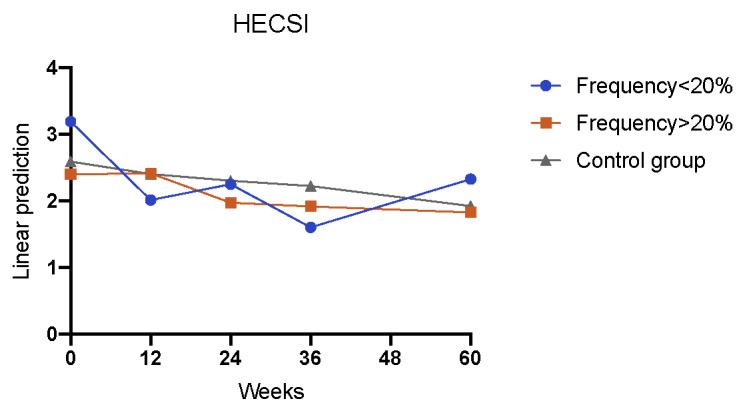
Development of HECSI regarding the control versus the intervention group with <20% frequency usage versus ≥20% usage frequency (observant=290; n=57; interaction week×<20%: week 12: *P*=.002; week 36: *P*=.002; week 60: *P*=.04). HECSI: Hand Eczema Severity Index.

Patients who used the app less frequently than once every 5 weeks exhibited a consistent and statistically significant reduction in HECSI scores from week 0 to week 60, except for week 24, in comparison with the control group (interaction week×<20%: week 12: Coef=–1.234; *P*=.002; week 24: Coef=–0.732; *P*=.06; week 36: Coef=–1.500; *P*=.002; week 60: Coef=–1.275; *P*=.04; Table 5, model 0; Figure 8). Throughout the 60-week study period, no significant reduction in HECSI scores was found among patients who used the app more frequently than once every 5 weeks (Table 3, model 0; Figure 8).

**Figure 9:**
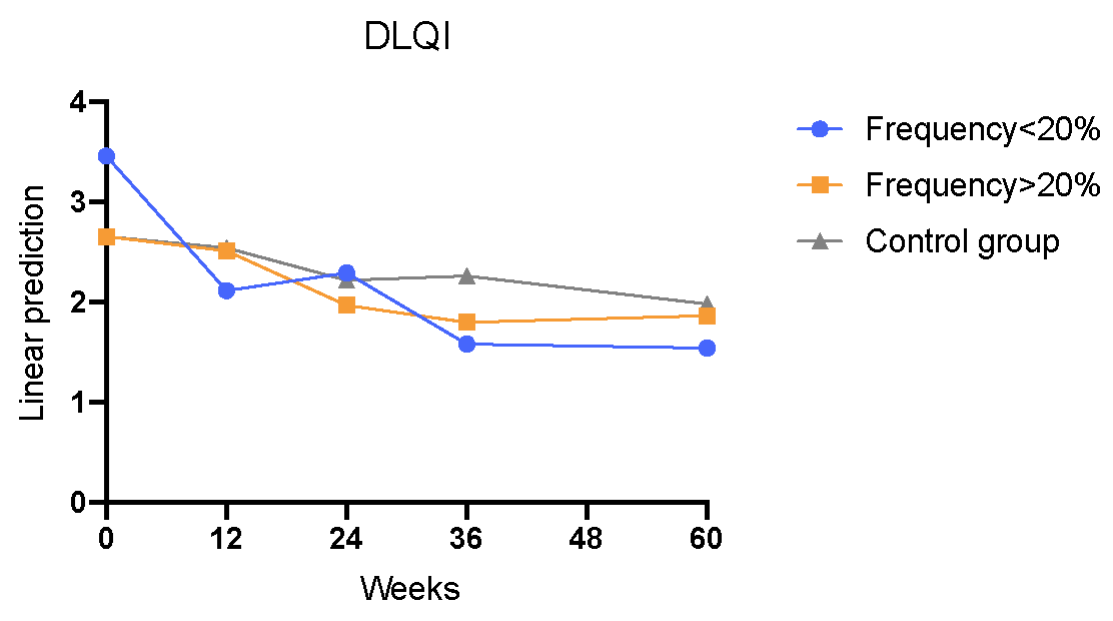
Development of DLQI regarding the control versus the intervention group with <20% usage frequency versus ≥20% usage frequency (observant=290; n= 57; interaction week×<20%: week 12: *P*=.002; week 36: *P*=.002; week 60: *P*=.04). DLQI: Dermatology Life Quality Index.

In addition, patients with an app usage frequency of less than 20% demonstrated a consistently significant reduction in DLQI scores throughout the entire study period, except for week 24. From the beginning to week 60, there was a significant improvement in DLQI scores (interaction week×<20%; week 12: Coef=–1.234; *P*=.002; week 24: Coef=–0.732; *P*=.06; week 36: Coef=–1.485; *P*=.002; week 60: Coef=–1.246; *P*=.04; Table 5, model 1; Figure 9). On the other side, no significant reduction was noticed in the group with app frequency of more than 20% (Table 5, model 1; Figure 9). Results were dependent on gender, age, and disease duration (Table 5, model 1).

A significant improvement in mood was also observed in patients with less than 20% app usage frequency at weeks 12 and 36 (interaction week×<20%; week 12: Coef=–0.990; *P*=.03; week 36: Coef=–1.289; *P*=.02; Table 5, model 0), consistent with the DLQI results.

No significant effects on activity and itching were noticed in patients with an app usage frequency of less or more than 20% (Table 5).

### Comparison of Data Collected From the DLQI and HECSI Through the App Compared to Those Collected During Personal Visits

The intervention group was asked to upload a picture of the affected area on hands and feet once a week. Doctors then calculated the eHECSI based on the uploaded pictures. The eHECSI was compared to the HECSI documented in personal visits. The same applied to the DLQI and electronic Dermatology Life Quality Index (eDLQI).

The eHECSI was comparable with the HECSI obtained during in-person visits (ρ=0.885; *P*≤.001; Multimedia Appendix 2). The same was true for the DLQI as the DLQI and the eDLQI showed a strong correlation (ρ=0.474; *P*≤.001; Multimedia Appendix 2). The assessment involved evaluating image quality in terms of lighting, sharpness or focus, and completeness. Despite the presence of suboptimal image quality, the calculated eHECSI still demonstrated a strong correlation with the in-person assessed HECSI (for good quality: ρ=0.884; *P*<.001; for bad quality: ρ=0.901; *P*≤.001; Multimedia Appendix 3).

## Discussion

### Principal Findings

In this study, we investigated the effect of an educational program combined with a smartphone app on the clinical outcome and well-being of patients with chronic hand and foot eczema. Results of the interim analysis at week 24 have already been published and demonstrate the efficacy of our intervention in terms of improvements in patients’ quality of life, pain levels, activity, and clinical outcomes [10]. The 60-week data presented here showed that the positive effects, particularly regarding quality of life and, to a lesser extent, clinical outcome, were maintained over 60 weeks. Therefore, the educational program combined with the app, which facilitates communication with the doctor through the chat function, seems to have positive long-lasting effects. The intervention may help patients cope better with the disease, understand it better, and ultimately adhere to treatment regimens. That educational programs lead to better outcomes in patients with hand eczema, is not new. Corti et al [12] retrospectively analyzed the records of 36 patients who participated in an educational program and showed that 67% (24/36) of patients with hand eczema benefited from the intervention, mainly due to behavioral changes regarding skin care and protection [12]. However, the follow-up visit was after 12 weeks, so long-term effects were not analyzed.

In our study, improvement in pain, activity, HECSI, and DLQI were observed in the whole study population. The improvement observed in both groups may be study related to the more intensive care of the patients by their dermatologists. Stewart et al [13] showed that close doctor-patient contact and good communication between both parties have positive healing effects [13], which underlines the importance of the control group when evaluating the effect of an intervention such as an educational program or an eHealth device.

Regarding the frequency of use of the app, we were able to show that less frequent use of the app resulted in better outcomes, mood, and quality of life. The reasons for this unexpected finding may be multifaceted. First, patients may not want to be confronted with their disease on a regular basis. Similar results were observed in our previous study, which was a 60-week monitoring app intervention study focused on assessing patients with psoriasis. In the group that used the app less than once every 5 weeks, patients showed significant improvement in depression and anxiety symptoms as measured by the Hospital Anxiety and Depression Scale. The conclusion of this study highlighted the preference of patients with chronic diseases to avoid frequent reminders of their conditions [11]. We recently held a workshop with patients with psoriasis and asked them what features an app should have to make it worth using (unpublished data). Interestingly, one of the most important pieces of feedback was that the app should help save time in health care. Patients do not want to spend time filling out long questionnaires or documenting their disease but want an app that can be used according to their health needs. This should be considered when developing future patient-centered apps.

Interestingly, gender and age had an impact on the frequency of app use; especially older women were most likely to use the app. There could be several reasons for this observation. The fact that older people use the app more often may be explained by the fact that they have more free time than younger people who are still pursuing their careers. The paper by Rosales and Fernández-Ardèvol [14] examines the smartphone usage and app preferences of older individuals residing in Spain. Their findings indicate that older adults tend to use smartphones and smartphone apps in a more utilitarian manner. Specifically, they frequently access personal information manager apps, such as calendars, address books, and notes, more often than younger age groups. This highlights how older users prioritize and use apps that serve practical purposes in their daily lives [14]. Older women may have a higher motivation for diligent self-monitoring of their condition, leading them to use the app more regularly. It would also be interesting to investigate whether the design or usability of the app specifically met the needs of older women, which might explain their higher usage. Further research and analysis could contribute to a better understanding of this result and provide valuable insights for future studies and developments. In terms of gender differences, the study by Graziano et al [15] found notable gender differences in the relationship between parent-reported self-regulation skills and adolescents’ management of type 1 diabetes. Specifically, among boys, it was observed that deficits in executive functioning and emotion regulation were significantly linked to poorer treatment adherence and glycemic control [15]. In another study examining the development of self-regulation in the first 4 years of life, it was found that girls exhibited higher levels of committed compliance compared to boys [16]. In general, there seem to be significant gender differences in the use of eHealth devices, which is also an important fact to consider in the development of health-related apps and needs further investigation.

In comparing the eHECSI obtained through photo documentation through the app with the in-person assessments during regular visits, we showed that the image quality uploaded in the app did not impact the objective assessment. Consistent results were found for both the eHECSI and the HECSI assessed in person. This suggests that even in cases where the image quality was not ideal, possibly due to the patient’s camera or photography technique, the HECSI could still be effectively assessed. This is certainly not true for all dermatologic conditions, especially those that can affect the entire body, and highlights that hand and foot eczema is a perfect candidate for teledermatologic monitoring. The implementation of a monitoring app, possibly combined with artificial intelligence, could therefore have a huge socioeconomic impact, saving time not only for the patient but also for the health care system.

It is important to note that the dropout rate in the intervention group was relatively high (46.5%) compared to the control group (22.7%). The most important factor contributing to the high dropout rate in the intervention group was the educational program required of patients in the intervention group. At the beginning of the study, it was difficult to schedule a time for the intervention group to receive the training and introduction to the app. Some participants initially agreed to attend, but later either did not show up or canceled at short notice, especially since many participants lived several miles from the clinic, making attendance a significant challenge. Based on our experience, for future studies, online training sessions could potentially engage more participants who are unable to travel long distances or have only a few minutes to attend the training from home.

### Limitations

The main limitations of our study are that the cohort became smaller over time and there is an imbalance in the number of patients in the groups. In particular, the number of patients in the intervention group became small over the 60 weeks of care, which may lead to misinterpretation of the results and differences between the 2 groups and the subgroup analysis. In addition, the patients in our study tended to be older, which means that it may be difficult to extrapolate the results to all age groups. In the scope of our app-focused study, a noteworthy limitation lies in our inability to disentangle the distinct effects of the educational program and the app within the intervention group. The intertwined nature of these 2 interventions makes it challenging to attribute the observed outcomes solely to 1 component. Further research could delve deeper into this aspect, potentially conducting separate investigations to better understand the individual contributions, interactions, and impact of the educational program and the app on our intervention group’s outcomes. Further studies are needed to verify our results and findings in a larger patient population.

### Conclusion

In summary, the combination of the educational program and the app had a clear positive influence on patients’ mental well-being and disease severity, particularly when the app usage frequency was moderate. It is worth noting that the app, when used in conjunction with in-person visits, contributed to the observed positive effects. Therefore, the app should be viewed as a supplementary tool and not a complete replacement for real-time medical consultations. As shown in the study by Baldwin et al [17], telemedicine can lead to a 75% reduction in outpatient clinic visits saving time for both patients and health care institutions. Teledermatology therefore offers the potential for a cost-effective supplementary health care approach, which should be integrated into the daily care of dermatological patients, especially in easy-to-monitor dermatological conditions such as hand and foot eczema [18].

## Data Availability

The datasets generated during and/or analyzed during this study are available from the corresponding author on reasonable request.

## Appendix

Multimedia Appendix 1 CONSORT-EHEALTH (Consolidated Standards of Reporting Trials of Electronic and Mobile Health Applications and Online Telehealth) checklist.

Multimedia Appendix 2 Spearman correlation comparing the electronic Hand Eczema Severity Index and electronic Dermatology Life Quality Index from the app with the Hand Eczema Severity Index and Dermatology Life Quality Index from in-person visits (observant=61).

Multimedia Appendix 3 Spearman correlation comparing the results of electronic Hand Eczema Severity Index and Hand Eczema Severity Index based on the quality of the picture downloaded through the app (observant=51).

## Abbreviations

Coef: Linear regression coefficient
CONSORT-EHEALTH: Consolidated Standards of Reporting Trials of Electronic and Mobile Health Applications and Online Telehealth
DiGA: Digital Health Applications
DLQI: Dermatology Life Quality Index
eDLQI: electronic Dermatology Life Quality Index
eHECSI: electronic Hand Eczema Severity Index
HECSI: Hand Eczema Severity Index
