# Supplementary material for: Improving Outcomes and Quality of Life for Patients With Hand and Foot Eczema: Randomized Study of a Patient-Centered Monitoring App
Source: J Med Internet Res. 2025 Jan 21;27:e52159. doi: 10.2196/52159 (PMC11795157; doi:10.2196/52159)
Supplement: Multimedia Appendix 2 [file jmir_v27i1e52159_app2.docx]

**Multimedia Appendix 2**. Spearman correlation comparing the eHECSI and eDLQI from the app with the HECSI and DLQI from in-person visits (observant=61).

| Correlation of eHECSI/eDLQI with HECSI/DLQI | | HECSI^a^ | DLQI^b^ |  |
| --- | --- | --- | --- | --- |
| **eHECSI^c^** | |  |  |  |
|  | Spearman rho | 0.885 | 0.357 | |
|  | Significant level | **<.001** | **.004** | |
| **eDLQI^d^** | |  |  |  |
|  | Spearman rho | 0.363 | 0.474 | |
|  | Significant level | **.004** | **.001** | |

^a^HECSI: Hand Eczema Severity Index based on the pictures from the app.

^b^DLQI: Dermatology Life Quality Index raised from the patients in the app.

^c^eHECSI: electronic Hand Eczema Severity Index based on the pictures from the app.

^d^eDLQI: electronic Dermatology Life Quality Index raised from the patients in the app.
