# Supplementary material for: Improving Outcomes and Quality of Life for Patients With Hand and Foot Eczema: Randomized Study of a Patient-Centered Monitoring App
Source: J Med Internet Res. 2025 Jan 21;27:e52159. doi: 10.2196/52159 (PMC11795157; doi:10.2196/52159)
Supplement: Multimedia Appendix 3 [file jmir_v27i1e52159_app3.docx]

**Multimedia Appendix 3.** Spearman correlation comparing the results of eHECSI and HECSI based on the quality of the picture downloaded through the app (observant=51).

| <insert column head here> | | HECSI^a^ |  |
| --- | --- | --- | --- |
| **eHECSI^b^ (good quality)** | |  |  |
|  | Spearman rho | 0.884 | |
|  | Significant level | **<.001** | |
| **eHECSI (bad quality)** | |  |  |
|  | Spearman rho | 0.901 | |
|  | Significant level | **<.001** | |

^a^HECSI: Hand eczema Severity Index based on the pictures from the app.

^b^eHECSI: Electronic Hand Eczema Severity Index based on the pictures from the app.
